# Trend in primary caesarean delivery: a five-year experience in ABRUZZO, ITALY

**DOI:** 10.1186/s12913-018-3332-2

**Published:** 2018-07-03

**Authors:** Pamela Di Giovanni, Tonia Garzarella, Giuseppe Di Martino, Francesco Saverio Schioppa, Ferdinando Romano, Tommaso Staniscia

**Affiliations:** 1 Department of Pharmacy, “G. d’Annunzio” University of Chieti-Pescara, via dei Vestini, 31 -, 66100 Chieti, Italy; 2 Postgraduate School of Public Health and Preventive Medicine, “G. d’Annunzio” University of Chieti-Pescara, via dei Vestini, 31 -, 66100 Chieti, Italy; 3 Department of Medicine and Aging, “G. d’Annunzio” University of Chieti-Pescara, via dei Vestini, 31 -, 66100 Chieti, Italy; 4 Department of Public Health and Infectious Diseases, “La Sapienza” University of Rome, Piazzale Aldo Moro, 5 –, 00100 Rome, Italy

**Keywords:** Primary caesarean section, Trend, Quality of care, Discharge diagnosis, Appropriateness, Robson classification

## Abstract

**Background:**

Primary caesarean section (PCS) rate is one of the main indicators of quality of care suggested by the Italian Government. Hospital rankings are usually based on it, therefore lower rates reflect more appropriate clinical practice. The aim of this study is to describe a five-year trend of PCS rate in Abruzzo region from 2009 to 2013 and to examine the medical indications for this mode of delivery.

**Methods:**

Forty-five thousand one hundred forty-nine deliveries occurring from 2009 to 2013 were collected from all hospital discharge records (HDR) and analyzed. Among them we found 12,542 PCS. Odds ratios (ORs) with 95% confidence interval (95% CI) were estimated using logistic regression methods to evaluate the relationship between maternal risk factors and PCS in hospital over 1000 delivery/yrs.

**Results:**

The five-year PCS rate was 28.9%, with a decreasing trend from 31.4% in 2009 to 26.1% in 2013. Vasto Civil Hospital shows the lowest PCS rate (17.9% in 2013) among hospitals with a maximum of 1000 deliveries per year, while Pescara Civil Hospital shows the lowest PCS rate (25.4% in 2013) among hospitals with over 1000 deliveries per year. Women with major risk factors for cesarean section delivered more frequently in maternity units over 1000 delivery/yrs. Logistic regression analyses showed as diabetes, hypertension, twin pregnancy, fetal distress and preterm delivery were significant risk factors to deliver in unit over 1000 delivery/yrs. The most frequent (overall 66.6%) discharge diagnosis recorded in Hospital discharge records (HDR) is “Caesarean Delivery Without Indication”. 7.3% of PCS made in Abruzzo concerns women living in other Italian regions. 11.4% of PCS contains one of the indications to caesarean section (CS) that the Italian Guidelines consider appropriate.

**Conclusions:**

During the analyzed period, Abruzzo showed a decreasing, but still too high, PCS rate, compared to the limits fixed by the Italian Ministry of Health. Considering the limitation of this study, based on administrative data that are poor in clinical information, it is not possible to define the appropriateness of all caesarean sections.

## Background

Caesarean section (CS) is the most commonly performed surgical procedure in developed countries [1, 2] and its growing incidence has focused the attention on the causes and the possible strategies to reduce the CS rate. Considering the epidemic proportion of CS, the World Health Organization (WHO) indicates that a CS rate greater than 10–15% is not justifiable for any region of the World [3]. Primary caesarean section (PCS) rate is one of the main indicators of quality of care according to the Italian Government [4]. PCS is defined as caesarean section performed to women who have not had a previous cesarean delivery. Caesarean section rates show a wide variation across countries in the world, ranging from 0.4 to 40%, and a continuous rise in the trend has been observed during the past 30 years [5]. In Italy the proportion of this rate rose from 11% in 1980 to 37.5% in 2010 [6, 7], despite the recommendation given by the WHO [8] and the Italian Government to reduce the proportion of caesarean sections. The assumption is that lower rates reflect more appropriate clinical practice and general better performances in developed countries [9]. It is also known that the method of delivery may constitute one potentially modifiable risk factor of maternal mortality and morbidity. Several studies estimate the maternal mortality rate due to elective caesarean section between 2.84 [10] and 3.11 [11], compared with the mortality rate for vaginal delivery. Despite the raised maternal mortality due to CS in high income country such as Italy, this increase probably is small compared to that in low incomes country that may have limited facilities and high rates of maternal risk factors as HIV.

The regulation on the definition of hospital care quality, structural, technological, and quantitative standards edited by the Italian Ministry of Health for hospitals with over 1000 deliveries per year, suggests to keep the PCS rate below 25% [12]. The Agenas National Outcomes Program (PNE) 2014 showed a decrease of PCS rate during the last 5 years all over Italian territory, highlighting a lot of differences across regions [13]. The aim of this study is the description of the trend in PCS rate in Abruzzo from 2009 to 2013 and the examination of the medical indications for this mode of delivery.

## Methods

The study considered all the deliveries performed between January 1, 2009 and December 31, 2013 in fourteen hospitals of Abruzzo (region). Information is collected from all hospital discharge records (HDR), using the hospital information system. This system includes information about the demographic characteristics of patients, the diagnoses, and the procedures followed during the hospitalization coded using the International Classification of Disease, 9th Revision, Clinical Modification (ICD-9-CM). The HDR of all women between 10 and 55 years of age, who delivered in the fourteen Maternity Units of the region, were extracted and identified using the Diagnosis Related Group (DRG) codes 370 (Cesarean Section with CC), 371 (Cesarean Section without CC), 372 (Vaginal Delivery with Complicating Diagnoses), 373 (Vaginal Delivery without Complicating Diagnoses), 374 (Vaginal Delivery with Sterilization and / or D&C), 375 (Vaginal Delivery with O.R. procedure Except Sterilization and / or D&C) or the principal or secondary diagnostic codes, V27.xx or 640.xy-676.xy where y = 1 or 2, or the intervention codes 72.x, 73.2×, 73.5×, 73.6, 73.8, 73.9×, 74.0, 74.1, 74.2, 74.4 and 74.99. All mothers with one of the following discharge diagnoses were excluded: 656.4 (intrauterine death), V27.1 (single stillborn), V27.4 (twins, both stillborn), v27.7 (multiple birth, all stillborn) and 654.2 (previous caesarean section). Moreover, all women non-resident in Italy were excluded. A caesarean delivery (CD) was identified by DRG codes 370 and 371 or ICD-9-CM diagnosis code 669.7× or intervention codes 74.0, 74.1, 74.2, 74.4 and 74.99. The PCS rate is calculated as the number of women at their first CD over the number of women with no previous CD. The following socio-demographic variables were collected: maternal age, citizenship and marital status. Also the region of residence was considered to estimate the patient mobility towards Abruzzo for a PCS. In order to verify if CDs were properly done, we searched the hospital discharge records on the principal or secondary diagnostic codes to find the clinical indications issued by the Italian Guidelines on caesarean sections [4].

In Abruzzo the Maternity Units belong to four Local Health Authority (ASL): ASL Avezzano-Sulmona-L’Aquila, ASL Lanciano-Vasto-Chieti, ASL Pescara and ASL Teramo. One of the fourteen maternity units belongs to a private hospital (Avezzano) and, as Popoli Civil Hospital, performed deliveries only in 2009. Maternity units were stratified into two categories: under and over 1000 deliveries/yrs.

### Statistical analysis

The qualitative variables were summarized as frequency and proportion. Annual PCS rates were computed as the number of women at their first CD over the number of women with no previous CD. Pearson’s Chi-squared Test was performed to evaluate differences in frequencies distribution among categorical variables. Odds ratios (ORs) with 95% confidence interval (95% CI) were estimated using logistic regression methods to evaluate the relationship between maternal risk factors and PCS in hospital over 1000 delivery/yrs. All statistical tests were evaluated at an alpha level of 0.05. Statistical analysis were performed using IBM® SPSS Statistics v 20.0 software (SPSS Inc., Chicago, Illinois, USA).

## Results

Forty-five thousand one hundred forty-nine deliveries were analyzed including 12,542 PCS. Maternal characteristics are reported in Table 1. Significant statistical differences (*p* < 0.001) were founded stratifying maternity units by maternal characteristics. Particularly maternity units over 1000 delivery/yrs. showed a greater frequency of older women (age 35–39 and age > 39). The five-year PCS rate was 28.9%, highlighting a decreasing trend from 31.4% in 2009 to 26.1% in 2013, as reported in Table 2. The PCS rate of each hospital is also showed in Table 2: Penne Civil Hospital is the only one with a rising trend of PCS rate (from 35.6 to 43.0%). Among hospitals with a maximum of 1000 deliveries per year, Vasto Civil Hospital has the lowest PCS rate (17.9% in 2013). Considering the two hospitals with over 1000 deliveries per year, Pescara Civil Hospital shows the lowest PCS rate (25.4% in 2013 vs. 31.3% in 2013 of Chieti Civil Hospital). The five-year PCS rate was stratified by maternal characteristics, showing significant differences, as reported in Table 3. Particularly the proportion of women aged over 35 years increased during the five-years period. Also unmarried and separated women increased during the study period. The proportion of Italian women decreased significantly (*p* = 0.007).

**Table 1:** Number of Primary Cesarean Section stratified by maternal characteristics

Maternal characteristics	Total n (%)	Maternity units over 1000 deliveries/yrs n (%)	Maternity units under 1000 deliveries/yrs n (%)	*p-value*
Age (yrs)				< 0.001
< 20	126 (1.0)	39 (0.9)	87 (1.1)	
20–24	986 (7.9)	270 (6.1)	716 (8.8)	
25–29	2517 (20.1)	776 (17.6)	1741 (21.4)	
30–34	4296 (34.3)	1476 (33.4)	2820 (34.7)	
35–39	3368 (26.9)	1323 (29.9)	2045 (25.2)	
> 39	1249 (10.0)	537 (12.1)	712 (8.8)	
Marital status				< 0.001
Unmarried	2273 (18.1)	990 (22.4)	1283 (15.8)	
Married	5963 (47.5)	1971 (44.6)	3992 (49.2)	
Separated	56 (0.4)	20 (0.5)	36 (0.4)	
Divorced	53 (0.4)	25 (0.6)	28 (0.3)	
Widow	13 (0.1)	7 (0.2)	6 (0.1)	
Not declared	3193 (25.5)	1396 (31.6)	1797 (22.1)	
Missing	991 (7.9)	12 (0.3)	979 (12.1)	
Citizen				< 0.001
Italian	10,899 (86.9)	3963 (89.6)	6936 (85.4)	
Non Italian	1643 (13.1)	458 (10.4)	1185 (14.6)

**Table 2:** Primary Cesarean Section rate, 2009–2013, stratified by Maternity units

	2009 n (%)	2010 n (%)	2011 n (%)	2012 n (%)	2013 n (%)
Abruzzo – Overall	2735 (31.4)	2812 (31.7)	2520 (29.3)	2266 (26.1)	2209 (26.1)
Maternity units over 1000 deliveries/yrs					
Chieti	445 (37.2)	485 (38.8)	474 (35.8)	403 (31.9)	373 (31.3)
Pescara	539 (28.7)	545 (29.5)	375 (23.5)	341 (21.1)	441 (25.4)
Maternity units under 1000 deliveries/yrs					
Private Hospital^a^- Avezzano	220 (51.0)				
Sant’Omero	129 (28.7)	126 (34.3)	91 (27.0)	137 (22.5)	185 (26.4)
Ortona	148 (40.0)	176 (46.0)	159 (40.3)	124 (31.4)	95 (24.5)
Teramo	221 (25.8)	202 (22.9)	174 (19.5)	170 (22.8)	150 (21.8)
Lanciano	200 (25.0)	211 (27.2)	218 (28.7)	145 (22.9)	132 (22.4)
Penne	53 (35.6)	73 (39.7)	79 (38.9)	68 (38.4)	99 (43.0)
Atri	137 (29.3)	133 (27.5)	126 (31.9)	79 (30.7)	89 (26.6)
L’Aquila	159 (29.4)	229 (27.5)	312 (30.9)	312 (30.5)	255 (26.7)
Avezzano	162 (26.3)	302 (30.0)	226 (24.7)	190 (22.2)	212 (25.7)
Popoli^a^	37 (46.3)				
Sulmona	129 (44.9)	133 (40.4)	110 (37.8)	111 (34.9)	100 (38.3)
Vasto	180 (25.8)	231 (32.6)	214 (32.6)	225 (28.8)	120 (17.9)

^a^Hospitals that performed deliveries only in 2009

**Table 3:** Primary Cesarean Section rate, 2009–2013, stratified by maternal characteristics

	2009 n (%)	2010 n (%)	2011 n (%)	2012 n (%)	2013 n (%)	*p-value*
Age (yrs)						0.006
< 20	30 (1.1)	31 (1.1)	22 (0.9)	22 (1.0)	21 (1.0)	
20–24	241 (8.8)	211 (7.5)	199 (7.9)	180 (7.9)	155 (7.0)	
25–29	563 (20.6)	595 (21.2)	505 (20.0)	450 (19.9)	404 (18.3)	
30–34	949 (34.7)	971 (34.5)	891 (35.4)	752 (33.2)	734 (33.2)	
35–39	721 (26.4)	750 (26.7)	659 (26.2)	607 (26.8)	631 (28.6)	
> 39	232 (8.5)	254 (9.0)	244 (9.7)	255 (11.3)	264 (12.0)	
Marital status^a^						< 0.001
Married	1430 (52.3)	1307 (46.5)	1265 (50.2)	871 (38.4)	1090 (49.3)	
Unmarried	451 (16.5)	430 (15.3)	504 (20.0)	437 (19.3)	451 (20.4)	
Divorced	13 (0.5)	13 (0.5)	13 (0.5)	7 (0.3)	7 (0.3)	
Separated	7 (0.3)	14 (0.5)	11 (0.4)	10 (0.4)	14 (0.6)	
Widow	2 (0.1)	2 (0.1)	5 (0.1)	3 (0.1)	1 (0.1)	
Citizen						0.001
Italian	2425 (88.7)	2473 (87.9)	2178 (86.4)	1928 (85.1)	1895 (85.8)	
Non Italian	310 (11.3)	339 (12.1)	342 (13.6)	338 (14.9)	314 (14.2)	
Maternity Units						0.007
Over 1000 deliveries/yrs	984 (36.0)	1030 (36.6)	849 (33.7)	744 (32.8)	814 (36.8)	
Under 1000 deliveries/yrs	1751 (64.0)	1782 (63.4)	1671 (66.3)	1522 (67.2)	1395 (63.2)	

^a^Unknown Status was not taken into account in analysis and relatives data were not reported in table

Table 4 describes maternal risk factors distribution: it shows that women with major risk factors for cesarean section delivered more frequently in maternity units over 1000 delivery/yrs. Logistic regression analyses showed as diabetes, hypertension, twin pregnancy, fetal distress and preterm delivery were significant risk factors to deliver in unit over 1000 delivery/yrs.

**Table 4:** Maternal risk factors to deliver in maternity units over 1000 deliveries/yrs

Maternal risk factors	Total n (%)	Maternity units over 1000 deliveries/yrs n (%)	Maternity units under 1000 deliveries/yrs n (%)	*Odds Ratio (95% CI)*	*p-value*
Diabetes	239 (1.9)	123 (2.8)	116 (1.4)	1.99 (1.54–2.57)	< 0.001
Hypertension	437 (3.5)	205 (4.7)	232 (2.8)	1.67 (1.38–2.02)	< 0.001
Preeclampsia	279 (2.2)	102 (2.3)	177 (2.2)	1.07 (0.83–1.37)	*0.234*
Premature rupture of membranes	887 (7.1)	323 (7.3)	564 (6.9)	1.06 (0.92–1.23)	*0.206*
Twin pregnancy	426 (3.4)	265 (6.0)	161 (2.0)	3.18 (2.60–3.88)	< 0.001
Fetal distress	675 (5.4)	294 (6.7)	381 (4.7)	1.46 (1.24–1.71)	< 0.001
Preterm delivery	231 (1.8)	116 (2.6)	115 (1.4)	1.89 (1.46–2.45)	< 0.001

Table 5 shows the eleven principal discharge diagnosis codes sorted by frequency: the most frequent codes (overall 66.6%) are 669.70 and 669.71, both corresponding to “Caesarean Delivery Without Indication”. All the indications with an overall rate lower than 1% are summarized into “Other Diagnoses”.

**Table 5:** Principal discharge diagnoses

Discharge diagnosis	n (%)
Caesarean Delivery without indication	8356 (66.6)
Breech presentation	525 (4.2)
High head at term	387 (3.1)
Obstructed labor	321 (2.6)
Fetal distress	317 (2.5)
Abnormality in fetal heart rate or rhythm	253 (2.0)
Twin pregnancy	208 (1.7)
Primary uterine inertia	174 (1.4)
Fetopelvic disproportion	159 (1.3)
Premature rupture of membranes	130 (1.0)
Poor fetal growth	121 (1.0)
Others	1591 (12.7)

The 7.3% of PCS made in Abruzzo concerns women who lived in other Italian regions. Most of them came from Lazio (40.7%), Molise (23%) and Marche (9.5%), as showed in Table 6. One thousand four hundred twenty-eight HDR of PCS (11.4%) contain, as principal or secondary diagnostic codes, one of the indications to CD that is considered appropriate by the Italian Guidelines [4]. As showed in Table 7, the most frequent code corresponds to “breech presentation” (only 9.7% of PCS).

**Table 6:** Patient mobility towards Abruzzo for Primary Cesarean Section

Region of origin	n (%)
Lazio	373 (40.7)
Avezzano	164 (44.0)
L’Aquila	81 (21.7)
Others	128 (34.3)
Molise	215 (23.4)
Vasto	154 (71.6)
Others	61 (28.4)
Marche	87 (9.5)
Sant’Omero	33 (37.9)
Chieti	14 (16.1)
Others	40 (46.0)
Other Regions	242 (26.4)

**Table 7:** Appropriate indications for Cesarean section

Indications	n (%)
Breech presentation	1218 (9.7)
Active genital *Herpes simplex* in third trimester of pregnancy	2 (0.1)
Placenta previa	203 (1.6)
Previous uterine rupture or previous cesarean delivery with longitudinal incision	–
Estimated fetal weight of 4500 g or more in diabetic women	5 (0.1)
Total	1428 (11.4)

Hospital discharge records were searched on the principal or secondary diagnostic codes for the caesarean section clinical indications

## Discussion

The majority of women undergone to PCS were Italian citizens, married women, ranging from 30 to 34 years of age. Despite the decreasing trend of PCS rate from 31.4% in 2009 to 26.1% in 2013, in Abruzzo, this proportion remains over the threshold fixed by WHO [3, 8] and by the Italian Guidelines[4]. Concerning the hospitals estimating over 1000 deliveries per year, both Pescara and Chieti hospitals showed a decreasing trend: from 28.7 to 25.4% in Pescara, from 37.2 to 31.3% in Chieti. Despite that, in the last year of the analyzed period, Pescara hospital showed an increasing trend (from 21.1 to 25.4%). Among hospitals under 1000 deliveries per year, Sulmona (from 34.9% to 38.3), Avezzano (from 22.2 to 25.7%), Penne (from 38.4 to 43.0%), and Sant’Omero (from 22.5 to 26.4%) showed also a growing rate. In 2013, Pescara and Vasto hospitals are the only two Maternity Units with a PCS rate near the thresholds established by Italian Government[4], respectively 25.4 and 17.9%.

Concerning risk factors for CS, the analysis showed as diabetes, hypertension, twin pregnancy, fetal distress and preterm delivery were significant risk factors to deliver in maternity unit over 1000/yrs. These results can be explained by the higher level of specialization of maternity unit of Pescara and Chieti, which follows high-risk women during all pregnancy period. Despite lower frequency of high-risk pregnancy, maternity units under 1000 deliveries/yrs. showed PCS rate over the threshold established by Italian Government [4].

The most frequent discharge diagnosis recorded in HDR is CD without Indication. This lack of clinical indications for CS may be attributable to HDR miscoding [14], that is, reasons for caesareans were not available or not reported through data sources or there was a real absence of maternal or fetal morbidity. In the last case, women underwent CS for their own explicit request or for internal organization of each Maternity Unit. It is known that the frequency of CS at maternal request is increasing all over Europe [15]. Several studies estimate the PCS rate among women with no medical or obstetrical indication at between 3 and 7% [16–19]. Although lack of information in data sources justifies the use of caesarean section, it is plausible that the PCS rate among women with no medical or obstetrical indication in Abruzzo is higher than the rate estimated by the mentioned studies.

Considering that the HDR system lacks medical information such as drugs therapy, parity, chorionicity and amnionicity in twin pregnancy, and details about the timing of delivery (elective or emergency procedure), it was not possible to analyze the appropriateness of indications of all PCS according to the Italian Guidelines [4]. Considering the limitation of our research, only the 11.4% of PCS seems to be appropriate (Table 7). Particularly, ICD coding system lacks of information about maternal request of CS or other non-medical indication, missing many cause of CS and classifying them as “CS without indications”. For the same reasons, it was not possible to stratify PCS according to Robson 10-Group Classification System [3, 20, 21].

## Conclusions

In the analyzed period, Abruzzo showed a decreasing, but still too high, PCS rate, compared to the limit fixed by the Italian Ministry of Health.

Considering the limitation of this study, based on administrative data that are poor in clinical information, it is not possible to define the appropriateness of all caesarean sections.

Due to the lack of important information about surgical procedure, as timing or other clinical and non-clinical information, Italian Discharge Registry needs improvements to allow growth in care policy.

## Abbreviations

CD: Caesarean delivery
CS: Caesarean section
DRG: Diagnosis related group
HDR: Hospital discharge records
ICD-9-CM: International classification of disease, 9th revision, clinical modification
PCS: Primary caesarean section
PNE: National Outcomes Program
WHO: World Health Organization
